# The hydrolysis of proteins by microwave energy

**DOI:** 10.1155/S1463924691000172

**Published:** 1991

**Authors:** Sam A. Margolis, Lois Jassie, H. M. Kingston

**Affiliations:** National Institute of Standards and Technology Center for Analytical Chemistry Organic Analytical Research Division Gaithersburg Maryland 20899 USA

## Abstract

Microwave energy, at manually-adjusted, partial power settings has been used to hydrolyse bovine serum albumin at 125 °C. Hydrolysis was complete within 2 h, except for valine and isoleucine which were completely liberated within 4 h. The aminoacid destruction was less than that observed at similar hydrolysis conditions with other methods and complete hydrolysis was achieved more rapidly. These results provide a basis for automating the process of amino-acid hydrolysis.


Journal of Automatic Chemistry, Vol. 13, No. 3 (May–June 1991), pp. 93–95

### The hydrolysis of proteins by microwave energy

**Sam A. Margolis, Lois Jassie and H. M. Kingston**

*National Institute of Standards and Technology, Center for Analytical Chemistry, Organic Analytical Research Division, Gaithersburg, Maryland 20899, USA*

*Microwave energy, at manually-adjusted, partial power settings has been used to hydrolyse bovine serum albumin at 125 °C. Hydrolysis was complete within 2 h, except for valine and isoleucine which were completely liberated within 4 h. The amino-acid destruction was less than that observed at similar hydrolysis conditions with other methods and complete hydrolysis was achieved more rapidly. These results provide a basis for automating the process of amino-acid hydrolysis.*

### Introduction

Microwave energy comprise waves of 1 mm to 100 m and is found between the radio and the infra-red regions of the electromagnetic spectrum. In aqueous or dielectric media, the microwaves may induce polarization in susceptible molecules, as well as promote the rotation of true molecular dipoles at rates approaching $2{\cdot}45 \times 10^9$ MHz, the frequency of the microwave commonly used for commercial microwave ovens. If the dielectric relaxation of the material is the same as the frequency of the radiation ($10^9$ to $10^{12}$ cycles/s), then the microwave radiation will couple directly with the oscillating particles in solution. The heat produced by microwave irradiation arises from two effects: (1) the frictional forces which result as the molecular dipoles align themselves with the passing wave front; and (2) the movement of hydrated charged ions or molecules with a dipolar solvent shell toward their oppositely charged poles.

Amino-acids, peptides and proteins are charged molecules which have dipolar properties. Dipole moments of the ethyl esters of selected amino-acids are approximately 2·1 Debyes, and those of selected amides range from 3·5 to 5 Debyes [1]. A protein is composed of a number of dipolar moieties which contribute to the net dipole moment of the total protein. This is further modified by the hydration shell of the molecule. It is conceivable that the electromagnetic energy could increase the rotational force on bonds connecting dipolar moieties to adjacent atoms, thereby reducing the energy required to break bonds and increasing the probability for the occurrence of such processes as hydrolysis.

The production of heat by microwave radiation (2450 MHz) of electrolyte solutions, such as concentrated HCl, which contain small amounts of organic materials is almost exclusively the result of the action of the microwave on the major electrolytes and not on the organic material. The combination of the heating effect and the possible increase in rotational forces on the peptide bonds may catalyse more effectively the hydrolysis of the peptide bonds and preserve the integrity of the less stable amino-acids.

The hydrolysis of proteins and peptides by microwave energy has been demonstrated under conditions where the microwave power was not controlled [2,3]. The heat-sensitive amino-acids, such as serine, threonine and tyrosine, showed a significant degradation (20, 40 and 35% respectively) [2], which is characteristic for exposure of these amino-acids to temperatures between 172 and 174 °C. The objective of this study is to evaluate the utility of using *controlled* microwave power as a procedure for hydrolysing proteins to their constituent amino-acids.

### Methods

### *Reagents*

The bovine serum albumin (BSA) used in this study was NIST Standard Reference Material (SRM) 926 [4]. High-purity water was prepared from distilled deionized water by subboiling distillation in a quartz apparatus. In a similar quartz still, 10 mol/l HCl was prepared by subboiling distillation of concentrated reagent grade HCl. Amino-acid calibration standards were obtained from Pierce (Rockford, Illinois). 9-Fluorenylmethoxy-carbonylchloride (FMOC) was obtained from Fluka (Ronkonkoma, New York, USA).

Duplicate BSA samples were prepared by adding 4·0 ml of an aqueous solution of albumin SRM 926 (16·05 mg/ml) to a clean acid-leached Teflon (PFA) pressure vessel which was free of metal impurities. Prior to the addition of the albumin solution, 1·5 ml of water and 6 ml of 10 mol/l HCl were added to the vessel. The vessels were flushed with nitrogen, tightly sealed and placed on a carousel in a laboratory microwave system [5]. One vessel in each experiment was fitted with a pressure sensor and a fibre-optic thermometer suitable for use in microwave instruments [5]. Temperature and pressure equilibration was achieved in 5 min and was constantly monitored with microwave power (145–98 W) being adjusted manually. The pressure varied between 0·75 and 0·96 atm (97 kPa), and the temperature between 124·6 and 125·2 °C.

Amino-acid analysis was performed and the amino-acid derivatives were prepared as described by Cunico *et al.* [6], except that an aliquot of base (1·2 mol/l NaOH) was added to an aliquot of either a standard mixture of amino-acids or a protein hydrolysate to neutralize the HCl. The FMOC amino-acids were analysed on a single pump liquid chromatograph with low pressure gradient mixing and measured with fluorescence spectrophotometer.

© Copyright U.S. Government 1991

### Results and discussion

The purpose of this study was to demonstrate that proteins can be hydrolysed by microwave radiation as rapidly, or more rapidly, than by classical methods [7], and with less destruction of the labile amino-acids, such as serine, threonine, and tyrosine. The results in table 1 demonstrate that hydrolysis by microwave energy is capable of achieving this objective. Bovine serum albumin SRM 926 was selected as a substrate for these studies, because its sequence is known [8–10], and because its purity and homogeneity have been well characterized for the purpose of using it as a clinical reference material for measuring serum proteins. The temperature of 125 °C was selected for microwave hydrolysis in order to permit the completion of the hydrolysis step within 4 h and to be able to compare these results with those obtained at 2 and 4 h at 145 °C [7].

*Table 1. Effect of microwave hydrolysis duration and amino-acid standard on the amino-acid composition of bovine serum albumin.*

| Amino-acid | Number of residues* Hydrolysis time 2 h | 4 h | Theory |
|---|---|---|---|
| Arg | 23 | 23 | 23 |
| Ser | 31 | 28 | 28 |
| Asp | 55 | 54 | 54 |
| Glu | 79 | 80 | 79 |
| Thr | 33 | 32 | 34 |
| Gly | 17 | 18 | 16 |
| Ala | 46 | 47 | 46 |
| Pro | 30 | 29 | 28 |
| Met | 4 | 4 | 4 |
| Val | 30 | 34 | 36 |
| Phe | 25 | 26 | 27 |
| Cystine | 14 | 16 | 17 |
| Ile | 11 | 13 | 14 |
| Leu | 59 | 62 | 61 |
| His | 18 | 19 | 17 |
| Lys | 58 | 59 | 59 |
| Tyr | 20 | 19 | 20 |

* Each value represents the mean value of the analysis of two separate hydrolysates calibrated against each of two independent standard amino-acid mixtures (a total of four measurements for each amino-acid at each time period).

Chen *et al.* [2] and Chiou *et al.* [3] did not regulate the microwave power in their studies and this led to degradation of the thermally labile amino-acids. In this study, the microwave power was manually regulated to maintain the temperature at 125 ± 2·1 °C based upon temperature measurements during the hydrolysis process. The control of the temperature in these experiments depends on reaching an energy equilibrium between applied microwave energy and heat loss from the sample vessel [5]. This form of control was not adequate to provide unattended operation of the system. Because some of the amino-acid residues are susceptible to degradation, manual adjustments were necessary to prevent temperature drift. Fully automated temperature feedback control has been implemented on a similar microwave unit to provide automated control for this type of biochemical hydrolysis in the future [11].

A comparison of the amino-acid composition of the sample hydrolysed for 2 h to that hydrolysed for 4 h indicates the hydrolysis was complete within the 2 h period, with the exception of a small percentage of valine, isoleucine and leucine residues the peptide bonds of which are resistant to hydrolysis [7]. Tryptophan was not measured because it is not stable to acid hydrolysis. Threonine, serine and tyrosine, which are partially degraded by other hydrolysis procedures [5,6,11,12], appeared to be stable. This stability was confirmed by parallel exposure of amino-acid mixtures to microwave radiation. These results indicate that microwave hydrolysis is less destructive than the classical hydrolysis procedure [7] and achieves complete hydrolysis at a lower temperature, in a given period of time. These results are comparable in precision and accuracy to those reported by other investigators using other methods of hydrolysis [6,7,12,13].

By the process of manual adjustment it is possible to control the microwave power on the basis of the observed temperature and thereby obtain reproducible hydrolysis conditions. This technique also provides the basis for designing a procedure for automating the process of protein hydrolysis and transferring this methodology to general laboratory use.

These results indicate for the first time that complete hydrolysis of proteins can be achieved by exposure to microwaves under controlled conditions. The hydrolysis can be carried out in a short time (2–4 h) at a relatively low temperature (125 °C). It appears that, in addition to the thermal effect, microwave radiation may induce or facilitate rupture of the peptide bond by a localized interaction with each polarizable amide group of the peptide chain.

### Note

Identification of any commercial products or equipment does not imply recommendation or endorsement by the National Institute of Standards and Technology, nor does it imply that the material or equipment identified is necessarily the best for the purpose.

### References

1. Weast, R. C., *Handbook of Chemistry and Physics* (The Chemical Rubber Co., Cleveland, 1972), p. E-54.
2. Chen, S., Chiou, S., Yen, Y. and Wang, K., *International Journal of Peptide and Protein Research,* **30** (1987), 572.
3. Chiou, S. and Wang, K., *Journal of Chromatography,* **491** (1989), 424.
4. Reeder, D. J. and Schaffer, R., In *Standard Reference Materials: Summary of the Clinical Laboratory Standards Issued by the National Bureau Of Standards*, edited by Seward, R. W. and Mavrodineanu, R. (U.S. Government Printing Office, Washington, D.C., 1981), NBS Special Publication 260–71.

5. Kingston, H. M. and Jassie, L. B., *Introduction to Microwave Sample Preparation: Theory and Practice* (American Chemical Society: Washington, D.C., 1988), Chapter 6.

6. Cunico, R., Mayer, A. G., Wehr, T. and Sheehan, T. L., *Biochromatography,* **1** (1986), 6.

7. Roach, D. and Gehrke, C. W., *Journal of Chromatography,* **52** (1970), 393.

8. Peters, T., *Clinical Chemistry,* **23** (1977), 5.

9. Wiejers, R. N. M., *Clinical Chemistry,* **23** (1977), 1362.

10. Ueno, A., Hong, Y. M., Arakaki, N. and Takeda, Y., *Journal of Biochemistry (Tokyo),* **98** (1985), 269.

11. Kingston, H. M. and Walter, P. J., in preparation.

12. Meltzer, N. M., Tous, G. I., Gruber, S. and Stein, S., *Analytical Biochemistry,* **160** (1987), 356.

13. Simpson, R. J., Neuberger, M. R. and Liu, T. Y., *Journal of Biological Chemistry,* **251** (1976), 1936.

